# Special Issue “Dental Health and Disease: From the Molecular and Pathological Perspectives”

**DOI:** 10.3390/ijms262411838

**Published:** 2025-12-08

**Authors:** Ancuta Goriuc, Ionut Luchian

**Affiliations:** Faculty of Dental Medicine, Grigore T. Popa University of Medicine and Pharmacy Iasi, 700115 Iasi, Romania

## 1. Introduction

Oral health extends beyond aesthetics; it represents an integral component of overall health, sustained through a delicate balance among host defense mechanisms, the oral microbiome, and environmental influences. Nowadays, the oral cavity is no longer viewed as a stand-alone entity but is part of a dynamic and complex biological system that connects local and systemic processes [1]. It hosts one of the most diverse microbial communities and acts as an active interface where molecular, immune and mechanical mechanisms constantly interact [2]. Moreover, diseases such as caries, periodontal disease or oral cancers are viewed as multifactorial disorders resulting from the complex interaction between the host’s immune system, the microbiome and the individual’s genetic susceptibility. Molecular biology, immunology, microbiology are areas in which dental research has expanded in recent years, profoundly changing the way we understand oral health and diseases [3]. Progress to date and future research will enable early diagnosis, more effective prevention, and personalized therapeutic interventions [4].

In this context, understanding dental health and disease from molecular and pathological perspectives is not just a research topic, but a true new scientific paradigm [5]. By integrating fundamental knowledge with pathological observations, researchers and clinicians can more clearly define the origin of diseases, identify relevant biomarkers, and develop targeted therapies. Ultimately, this integrated approach confirms a simple but essential reality: oral health cannot be separated from overall health, and molecular understanding is the key to maintaining both [4,6].

## 2. Overview of the Special Issue

The special issue “Dental Health and Disease: From the Molecular and Pathological Perspectives” brings together 19 articles, including 14 original research papers and 5 review articles, which explore the complexity of oral pathology from different but complementary angles. Together, these papers reflect the variety of the field and the interdisciplinary nature of modern dental research.

Broadly speaking, the papers published in this special issue addressed diverse topics such as inflammatory and immune mechanisms involved in the initiation of dental diseases, the role of biomarkers in the molecular diagnosis of periodontal diseases, biomaterials with an impact on the development and regeneration of dental tissues and, last but not least, the interconnection of the oral cavity and systemic diseases.

## 3. Highlights from the Published Articles

Several studies in this special issue focused on the molecular mechanisms involved in inflammation in periodontal disease and other oral cavity diseases. Piekoszewska-Ziętek et al. showed that elevated salivary levels of IL-6 and IL-18 may be associated with the inflammatory state specific to idiopathic nephrotic syndrome, suggesting that saliva could be considered a suitable non-invasive medium for identifying different proinflammatory biomarkers [contribution 1]. Ghemis et al. reviewed the involvement of resolvins in tissue repair mechanisms in periodontal disease associated with type 2 diabetes [contribution 2], and Rodean et al. identified an association between elevated levels of systemic inflammatory markers and the presence of periodontopathic bacteria in patients with acute coronary syndromes, suggesting a direct link between periodontal infection and cardiovascular damage [contribution 3].

Aravindraja et al. analyzed miRNA expression in *Streptococcus gordonii* infection, indicating that *S. gordonii* may have an active role in the initiation and progression of periodontal disease through epigenetically regulated molecular mechanisms [contribution 4].

The identification of reliable molecular markers is a constant research topic in dentistry. Dolińska et al. reviewed the progress in molecular diagnostics of periodontal diseases [contribution 5], and Salatino et al. used nanopore sequencing to identify epigenetic changes in molar-incisor hypomineralization [contribution 6]. All these studies reinforce the idea that saliva and oral tissues can become valuable sources for non-invasive diagnostics contribution [contribution 7,8].

Another interesting study in mice is that of Kobayashi et al. who showed that type 2 diabetes can affect stem cell differentiation during tooth formation [contribution 9]. On the other hand, Poblano-Pérez et al. demonstrated the immunomodulatory role of mesenchymal stem cells derived from dental tissues, and Novacescu et al. provided histological and molecular insights into human tooth morphogenesis—essential information for future regenerative strategies [contribution 10,11].

Several papers in this special issue have highlighted the bidirectional relationship between oral inflammation and general diseases [contribution 12,13]. For example, Rodean and colab. confirmed a direct link between periodontal pathogens and inflammation in cardiovascular diseases [contribution 3], and Łobacz et al. analyzed the molecular aspects of periodontitis and hematological diseases [contribution 14]. Muñoz-Carrillo et al. addressed the interaction between periodontitis, diabetes, and COVID-19, concluding that periodontal disease, type 2 diabetes, and SARS-CoV-2 infection are interconnected through a common mechanism of chronic inflammation and immunometabolic dysfunction, where each condition can exacerbate the severity of the other [contribution 15]. Labis et al. studied the role of metal nanoparticles in the process of salivary stone formation and concluded that they are active participants in the lithogenesis process, which gives them a potential pathogenic role and opens new directions for the prevention and control of stone formation through molecular and toxicological approaches [contribution 16].

Alexa et al. explore how essential oil–based formulations can limit biofilm formation on orthodontic archwires, blending molecular docking with laboratory testing to highlight a promising, nature-inspired approach to reducing bacterial accumulation [contribution 17]. Kametani et al. look at how eluates from surface pre-reacted glass ionomer fillers can inhibit *Streptococcus mutans* even in sugar-rich conditions, meanwhile [contribution 18], Teles and co-authors shed light on a practical but often overlooked issue—the contamination of gutta-percha points with *Staphylococcus aureus*—reinforcing how essential proper infection control remains in daily endodontic practice [contribution 19]. Together, these studies illustrate the field’s ongoing commitment to improving patient care through advanced materials, refined techniques, and a deeper understanding of microbial behavior.

## 4. Conclusions and Future Perspectives

The 19 articles in this special issue provide a coherent perspective of modern oral health research. They emphasize the bidirectional relationship between oral and systemic health and demonstrate how oral health reflects and influences systemic health while molecular and pathological research paves the way for precision dentistry based on biomarkers, tissue regeneration, and inflammation control.

The future of research in this field will focus on validating molecular biomarkers for clinical use, integrating technologies for disease prediction, and developing safe and effective regenerative therapies. Interdisciplinary collaboration—between dentists, molecular biologists, immunologists, and dental materials specialists—remains essential to transform molecular knowledge into concrete clinical benefits, thus opening real prospects for precision, preventive, and personalized dentistry.
